# ^161^Tb-PSMA radioligand therapy in prostate cancer: current evidence and future perspectives

**DOI:** 10.3389/fonc.2025.1743628

**Published:** 2026-01-14

**Authors:** Liming Xiao, Ziyi Zhao, Rui Luo, Fucen Liu, Bosen Hu, Peng Zhao, Xia Yang, Zhengguo Chen

**Affiliations:** 1 Department of Nuclear Medicine, Mianyang Central Hospital, Mianyang, China; 2 National Health Commission (NHC) Key Laboratory of Nuclear Technology Medical Transformation (Mianyang Central Hospital), Mianyang, China; 3 National Defense Technology College of Southwest University of Science and Technology, Mianyang, China; 4 Institute of Nuclear Physics and Chemistry, Chinese Academy of Engineering Physics, Mianyang, China; 5 Targeted Radiopharmaceuticals Creation Key Laboratory of Sichuan Province, Mianyang, China

**Keywords:** dosimetry, lutetium-177 (^177^Lu), metastatic castration-resistant prostate cancer(mCRPC), prostate-specific membrane antigen (PSMA), PSMA-targeted radioligand therapy (PRLT), radiopharmaceuticals, terbium-161 (^161^Tb)

## Abstract

Prostate-specific membrane antigen (PSMA), a type II transmembrane glycoprotein, is overexpressed on the membranes of prostate cancer cells. Lutetium-177 (^177^Lu)- labelled PSMA-targeted radioligand therapy (PRLT) is employed in treating metastatic castration-resistant prostate cancer (mCRPC) that no longer responds to conventional therapies. However, some patients develop resistance or exhibit limited responsiveness, resulting in disease progression. Terbium-161 (^161^Tb) shares physical properties with ^177^Lu, as both isotopes emit β^-^ particles. Notably, ^161^Tb also emits internal conversion and Auger electrons, offering potential advantages in the effective targeting of small lesions. This dual-emission mechanism enables the treatment of lesions of varying sizes, generating growing interest in ^161^Tb-labelled radioligand therapy for prostate cancer. This review summarizes current evidence on ^161^Tb-PSMA, including its mechanism of action, radiolabeling and quality-control procedures, dosimetry, preclinical results, and clinical outcomes, highlighting its therapeutic promise. Future investigations should further validate the safety and efficacy of ^161^Tb-PSMA radioligand therapy, while enhancing its accessibility and clinical translation.

## 1 Introduction

Prostate cancer (PCa) is the most prevalent malignant tumor of the male genitourinary system. According to GLOBOCAN 2022 data, PCa ranks second in global incidence and fifth in mortality among malignancies affecting men (1). In China, PCa is the sixth most commonly diagnosed cancer and the seventh leading cause of cancer-related death (2), with the number of new cases increasing annually. A significant proportion PCa cases are diagnosed at intermediate or advanced stages. Approximately 5%–15% PCa cases are metastatic at initial diagnosis; this proportion can increase to 20%–25% or even higher in countries with limited medical resources (3). Androgen deprivation therapy (ADT) is as a cornerstone in the treatment of newly diagnosed hormone-sensitive PCa (HSPC) and advanced PCa. ADT is utilized across all treatment stages of PCa, except for very-low-risk and low-risk early localized PCa, and plays a critical role, particularly in HSPC (4–6). Patients with metastatic PCa initially respond to ADT; however, 10%–20% patients with PCa progress to castration-resistant PCa (CRPC) within a 5-year follow-up period (7), and ultimately, nearly all patients progress to CRPC (8). Despite significant advances in therapy, the prognosis for individuals with metastatic castration-resistant PCa (mCRPC) remains poor, posing a major challenge in PCa management (9).

Over the past 2 decades, treatment options for mCRPC have expanded to include chemotherapy, next-generation endocrine therapies, poly (ADP-ribose) polymerase inhibitors, sipuleucel-T immunotherapy, and immune checkpoint inhibitors (5). These approaches have improved disease control and prolonged overall survival. Nevertheless, therapeutic options remain limited for patients whose disease progresses after standard treatments, underscoring the urgent need for novel therapeutic strategies.

In recent years, radioligand therapy (RLT) targeting prostate-specific membrane antigen (PSMA) has gained prominence as a treatment for mCRPC (10). Lutetium-177 (^177^Lu)- PSMA-617 RLT effectively prolongs progression-free survival, particularly in individuals with larger lesions. However, β^-^ particles emitted by ^177^Lu may be insufficient to eradicate microlesions due to their limited energy and short tissue penetration range, highlighting the need for radionuclides that can more effectively target microscopic disease. Terbium-161 (^161^Tb) is an emerging therapeutic radionuclide. Like ^161^Tb and ^177^Lu emit β^-^ particles, but ^161^Tb offers a distinct advantage through the additional emission of internal conversion and Auger electrons, enabling more effective elimination of microlesions (11). Consequently, the use of ^161^Tb has garnered growing interest.

## 2 Mechanism of PSMA theranostics

**Figure 1:**
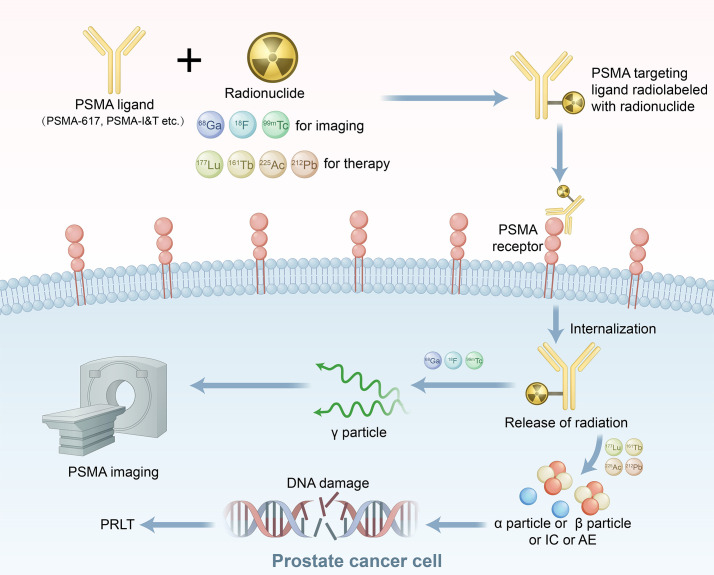
Mechanism of action for PSMA theranostics. AE, Auger electrons; IC, internal conversion electrons; PRLT, PSMA-targeted radioligand therapy; PSMA, prostate-specific membrane antigen.

PSMA, a type II transmembrane glycoprotein (12), is typically overexpressed in primary PCa and its metastases, with particularly high expression levels observed in mCRPC (13, 14). Consequently, PSMA represents an optimal molecular target for both diagnostic and therapeutic applications in mCRPC. PSMA ligands labelled with diagnostic radionuclides (^99m^Tc, ^18^F) and therapeutic radionuclides (^177^Lu, ^161^Tb), selectively bind to PSMA expressed on the extracellular membrane of PCa cells. These ligand–receptor complexes undergo internalization and are subsequently transported into the cells, where they release γ particles for PSMA-based imaging or emit α particles, β^-^ particles, internal conversion electrons, and Auger electrons that cause irreversible damage to critical biomolecules in tumor cells, such as double-strand DNA breaks, ultimately leading to PCa cell death (15) (**Figure 1**). Notably, PSMA expression is heterogeneous, particularly considering the effects of ADT on PSMA expression (16). Although the literature lacks consistency, most studies indicate that short-term ADT upregulates PSMA expression (16, 17). Consequently, ADT may affect the therapeutic outcomes of PSMA-targeted radioligand therapy (PRLT). Currently, second-generation antiandrogens are emerging as potential endoradiosensitizers in mCRPC patients (16). Some studies have found that ADT combined with PRLT can result in superior clinical outcomes (18–20).

## 3 Properties and advantages of ^161^Tb

**Figure 2:**
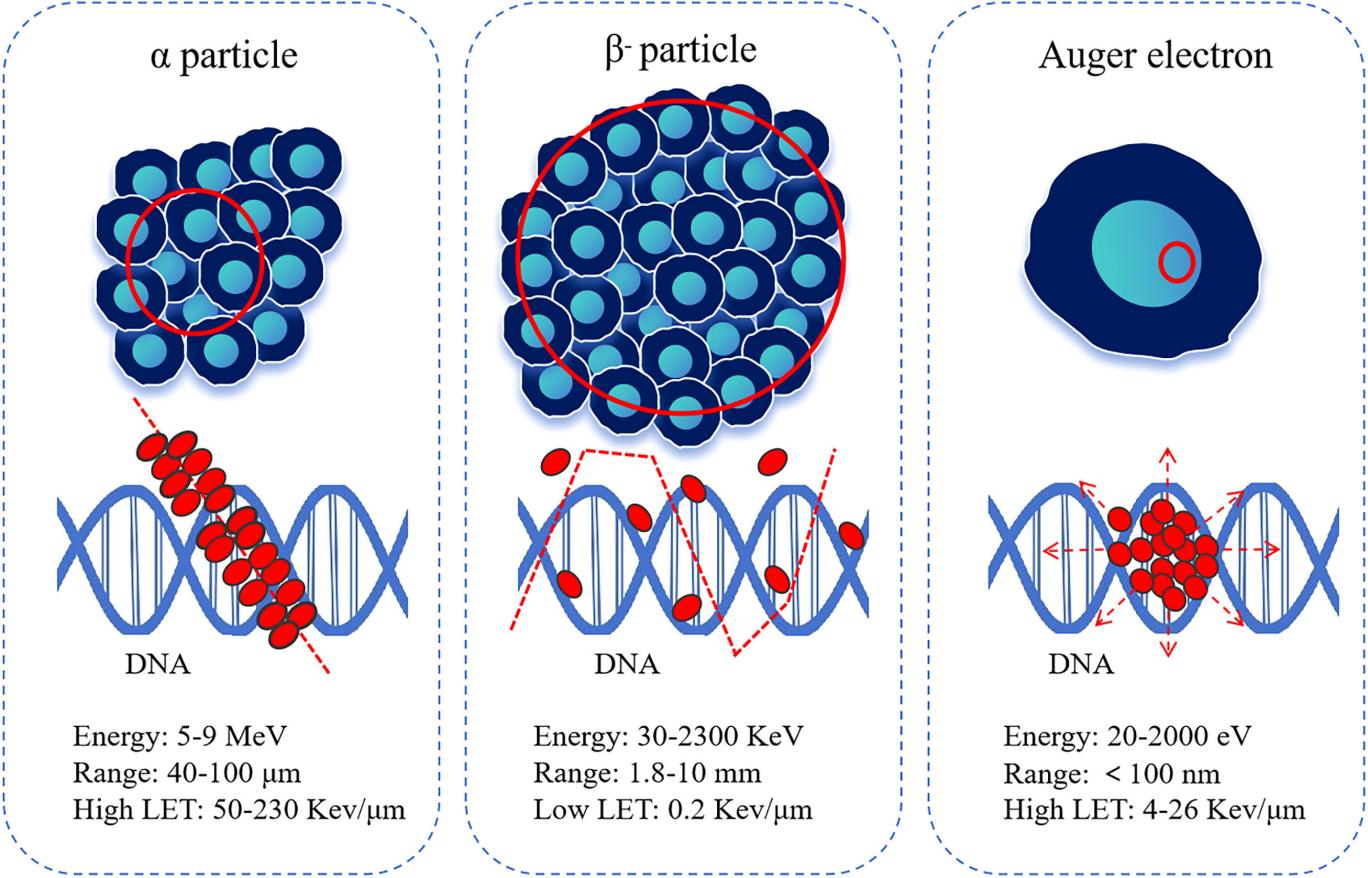
Nature and characteristics of different particles. LET, linear energy transfer.

While β^-^ particles remain the most established modality for RLT, increasing research attention has shifted toward α particles and Auger electrons to improve therapeutic efficacy. These particles possess shorter path lengths, higher linear energy transfer (LET), and greater relative biological effectiveness, resulting in enhanced cytotoxic potential and the capacity to overcome tumor cell resistance to radiopharmaceuticals (21–23) (**Figure 2**). Representative examples include Actinium-225 (^225^Ac), which emits α particles during decay (24), and ^161^Tb, which emits Auger electrons upon decay (25). ^161^Tb shares similar physical properties with ^177^Lu, including comparable half-life and β^-^ particle energies. The key distinction lies in its emission profile: ^161^Tb releases approximately 12.4 internal conversion and Auger electrons below 50 keV per decay. As a result, the total energy deposited per decay is about 202.5 keV for ^161^Tb, compared with 147.9 keV for ^177^Lu, indicating a higher overall energy delivery at the cellular level.

**Table 1** summarizes the decay characteristics of ^161^Tb and ^177^Lu (26, 27). Auger electrons are characterized by their ultra-short tissue penetration range (≤500 nm) and high LET of approximately 4–26 keV/μm. This combination markedly enhances the local radiation dose delivered to microscopic disease foci, enabling the effective eradication of circulating tumor cell clusters, micrometastases, and minimal residual disease (28, 29). The presence of such microscopic lesions is strongly associated with poor clinical outcomes in individuals with cancer (30). Consequently, ^161^Tb-PSMA holds considerable promise in the treatment of mCRPC.

**Table 1:** Decay characteristics of ^161^Tb and ^177^Lu.

Radionuclide	^161^Tb	^177^Lu
Half-life (days)	6.9	6.7
Type of decay (%)	β^-^ (100%)	β^-^ (100%)
Daughter nuclide	^161^Dy (stable)	^177^Hf (stable)
Average β^-^ energy (keV)	154.3	133.3
γ energy (KeV, % abundance)	25.7 keV (23%)	112.9 keV (6%)
	48.9 keV (17%)	208.4 keV (10%)
	74.6 keV (10%)	-
Energy per decay of ICE (keV)	39.3	13.5
Energy range of ICE (keV, weighted average)	3.3–98.3 (28)	6.2–206.3 (87)
Energy per decay of AE (keV)	8.9	1.1
Energy range of AE (keV, weighted average)	0.018–50.9 (0.8)	0.01–61.7 (1)
Total electron energy (keV)	202.5	147.9

ICE, Internal conversion electrons; AE, Auger electrons.

## 4 Production, radiolabeling, and quality control of ^161^Tb-PSMA radiopharmaceuticals

^161^Tb can be produced through two principal approaches: accelerator-based and reactor-based production. In the accelerator-based method, Nigron et al. (31) synthesized ^161^Tb via the gadolinium-160 (^160^Gd)(d,n) ^161^Tb reaction, achieving a yield of 10.3 MBq/μA/h and a radionuclidic purity of 86%. Similarly, Fedotova et al. (32) produced ^161^Tb via the ^nat^Dy(γ, pxn)^161^Tb reaction, yielding 14.4×10^3^ Bq·μA^−1^·h^−1^·cm^2^·g _Dy2O3_^−1^. However, accelerator-based production of ^161^Tb inevitably results in the formation of long-lived impurities, such as terbium-160 (^160^Tb) and other terbium or dysprosium isotopes. Without highly efficient separation technologies, accelerator methods are unlikely to be suitable for large-scale ^161^Tb production (31, 32). Alternatively, in reactor-based production,^161^Tb is generated via neutron irradiation of ^160^ Gd targets. Gracheva et al. (33) reported that a single irradiation cycle yielded ^161^Tb with a radioactive specific activity of 11–21 MBq/μL, radionuclidic purity ≥99%, and total activity up to 20 GBq. The resulting ^161^Tb-DOTATOC complexes exhibited excellent stability and high radiochemical purity, confirming their clinical suitability. **Table 2** summarizes the production characteristics and parameters of reactor-generated ^161^Tb (33–35). These findings indicate that reactor-based production supports scalable and consistent generation of ^161^Tb, providing a reliable source of medical isotopes for the development and clinical application of ^161^Tb-labelled radiopharmaceuticals.

**Table 2:** Characteristics and production parameters of reactor-produced ^161^Tb.

Reactor	Nuclear reaction type	Target material	Irradiation time (d)	Target material weight (mg)	Actual yield (GBq)
ILL	^160^Gd(n,γ)^161^Gd→^161^Tb	^160^Gd_2_O_3_	5	12.5	11.6
ILL		^160^Gd_2_O_3_	10	7.3	16.7
SAFARI-1		^160^Gd_2_O_3_	14	33.3	19.6
SAFARI-1		^160^Gd_2_O_3_	7	32.5	11.9
SINQ		^160^Gd_2_(NO_3_)_3_	21	94.9	8.8
SINQ		^160^Gd_2_(NO_3_)_3_	21	86.4	6.0
TRIGA		^160^Gd_2_O_3_	0.125	10.0~12.0	1.3~2.1×10^-3^
TRIGA		^nat^Gd_2_O_3_	0.125	50	4.3×10^-4^
ETRR-2		^nat^Gd_2_O_3_	0.5	100	8.9~9.8×10^-2^
ETRR-2		^nat^Gd_2_O_3_	1	100	1.9~2.0×10^-1^

Owing to the similar chemical properties of ^161^Tb and ^177^Lu, synthesis protocols established for ^177^Lu-based agents can be directly adapted for the preparation of ^161^Tb radiopharmaceuticals. This chemical compatibility enables the translational application of experience gained from ^177^Lu production, thereby accelerating the clinical adoption of ^161^Tb-labelled compounds (36). Established methods have been developed for the labelling and quality control of ^161^Tb-PSMA ligands. Müller et al. (23) prepared ^161^Tb-PSMA-617 by adding ^161^TbCl_3_ to a mixture containing PSMA-617, sodium acetate (0.5 M), and hydrochloric acid (0.05 M). The solution was incubated at 95°C for 10 min at pH 4.5, yielding ^161^Tb-PSMA-617 with a specific activity of 100 MBq/nmol. High-performance liquid chromatography (HPLC) confirmed a radiochemical purity of ≥ 98%. Radiolysis was observed after 1 h; however, the addition of L-ascorbic acid prevented decomposition. The formulation demonstrated excellent stability at 1 h, 4 h, and 24 h, with radiochemical purity maintained at ≥ 98%. Uygur et al. (37) developed and optimized an alternative labelling method. Specifically, 1 mL of sodium acetate buffer and 185 MBq of ^161^TbCl_3_ were added to a vial containing 50 μL of ascorbic acid. Following incubation of the reaction mixture (pH 4.5) at 95°C for 10 min, 25 μL of PSMA-617 was added. The mixture was then incubated again at 95°C for approximately 25 min and cooled to room temperature. Quality control using thin-layer chromatography and HPLC confirmed a radiochemical purity of 97.98% ± 2.01%, consistent with the 98% reported by Müller et al. (23). Stability testing showed that radiochemical purity remained >95% for up to 72 h. Subsequently, this team adapted this method to meet in-house Good Manufacturing Practice (GMP) compliance for first-in-human administration and evaluated it in a 77-year-old patient with mCRPC, demonstrating favorable biodistribution and urinary excretion (38).

## 5 Dosimetry of ^161^Tb-PSMA radiopharmaceuticals

Theoretical modelling indicates that ^161^Tb delivers higher radiation doses than ^177^Lu, particularly in micrometastases disease. In 2016, Hindié et al. (28) demonstrated, using Monte Carlo CELLDOSE simulations, that ^161^Tb deposits greater absorbed doses in spherical targets than ^177^Lu. This advantage arises not only from the β^-^ emissions of ^161^Tb being approximately 15% more energetic than those of ^177^Lu, but, more importantly, from the substantial contribution of Auger electrons emitted by ^161^Tb to the overall absorbed dose. The magnitude of this contribution is strongly dependent on tumor size. At the cellular or cell-cluster scale, the dose delivered to the nucleus—or to centrally located nuclei within clusters—by ^161^Tb exceeds that of ^177^Lu, regardless of whether the radionuclide is localized on the cell membrane, within the cytoplasm, or in the nucleus (29). Each medical radioisotope has an optimal curative range determined by lesion size. These calculations highlight that ^161^Tb offers a distinct advantage over ^177^Lu for targeting single tumor cells or micrometastases <1 mm, with the benefit becoming increasingly pronounced as lesion size decreases. However, due to intrinsic tumor heterogeneity, radionuclides are not uniformly distributed within all lesions, leading to spatial variations in absorbed dose.

To address this, Larouze et al. (29) in 2023 incorporated heterogeneity factors into absorbed-dose simulations for both ^177^Lu and ^161^Tb. Their results showed that ^161^Tb delivered a two- to three-fold higher radiation dose to the cell nucleus and a two- to six-fold higher dose to the cell membrane compared with ^177^Lu. Thus, even when accounting for intra-tumoral non-uniformity,^161^Tb may eradicate tumor cell clusters more effectively than ^177^Lu (30). Verburg et al. (26) reported similar findings using OLINDA V2.2.3 dosimetry software and an adult anthropomorphic model based on clinical data from ^177^Lu-PSMA-617. When substituting ^177^Lu with ^161^Tb, the results indicated a 40% increase in the absorbed dose per unit activity for ^161^Tb compared with ^177^Lu. Bernhardt et al. (39) employed a dynamic metastatic progression model to evaluate the therapeutic potential of ^161^Tb in disseminated PCa. Their modelling estimated the absorbed dose required to achieve metastatic disease control and revealed that ^161^Tb-PSMA ligands exhibited the greatest potential to enhance treatment response rates in advanced metastatic PCa. The low-energy electron emissions of ^161^Tb provide a distinct radiobiological advantage for the selective targeting of small tumors and micro-metastases.

## 6 Preclinical studies of ^161^Tb-PRLT

Both cellular and animal studies have demonstrated that ^161^Tb-labelled radiopharmaceuticals are more effective than ^177^Lu-labelled agents in suppressing tumor cell viability. Early preclinical investigations conducted in 1995 confirmed the feasibility of ^161^Tb for intraoperative imaging and radionuclide therapy applications (40). Subsequent animal studies comparing ^177^Lu and ^161^Tb-based agents revealed superior inhibition of tumor cell viability by ^161^Tb (41–43). Tschan et al. (44) compared the dosimetry and therapeutic efficacy of ^161^Tb and ^177^Lu-labelled PSMA ligands in mice with PCa.^161^Tb/^177^Lu-SibuDAB exhibited longer blood retention and higher tumor uptake than ^161^Tb/^177^Lu-PSMA-I&T. Notably, the tumor-absorbed dose for ^161^Tb-SibuDAB was approximately fourfold higher than that of ^161^Tb-PSMA-I&T, resulting in markedly improved tumor growth suppression without nephrotoxicity or other adverse effects. Compared with ^177^Lu, ^161^Tb increased the tumor radiation dose by about 40%.

Another preclinical study using PCa cell lines and xenograft models demonstrated that, at equivalent activity levels,^161^Tb -PSMA-617 significantly reduced PC-3PIP cell viability and survival compared with ^177^Lu-PSMA-617. Statistically significant differences (p <0.05 for both) were observed across activity concentrations of 0.1–10 MBq/mL and 0.05–5.0 MBq/mL. Moreover, mice treated with ^161^Tb-PSMA-617 exhibited dose-dependent increases in median survival—36 days and 65 days at injected activities of 5.0 MBq/mouse and 10 MBq/mouse, respectively—compared with 19 days in untreated controls. Collectively, these findings confirm that ^161^Tb-PSMA-617 provides superior *in vitro* and *in vivo* efficacy relative to ^177^Lu-PSMA-617, consistent with theoretical dose calculations indicating an additive therapeutic contribution from internal conversion and Auger electrons emitted by ^161^Tb (23).

## 7 Clinical studies of ^161^Tb-PRLT

In 2023, the Nuclear Medicine team at Saarland University Medical Centre, Germany, evaluated the therapeutic efficacy of ^161^Tb-PSMA-617 in patients with advanced mCRPC. One patient who had progressed after treatment with ^177^Lu-PSMA-617 exhibited markedly reduced PSA levels, decreased tumor burden, and alleviation of bone pain following ^161^Tb-PSMA-617 therapy. These findings suggest that ^161^Tb-PSMA-617 holds significant promise for treating mCRPC, particularly in patients for whom ^177^Lu-PSMA-617 therapy has failed (45). In the same year, the Nuclear Medicine team at the King Hussein Cancer Centre in Amman, Jordan, reported the first human SPECT imaging results following ^161^Tb-PSMA RLT. A 69-year-old patient with mCRPC received 5,550 MBq of ^161^Tb-PSMA-617 and tolerated the treatment well, with no acute or early adverse effects reported. Whole-body planar and SPECT/CT imaging were performed to obtain time–activity distribution data for ^161^Tb-PSMA-617 in tumor lesions and dose-limiting organs (46).

In 2024, the Saarland University Medical Centre conducted a clinical study involving six patients with mCRPC who had previously received ^177^Lu-PSMA-617 or combination therapy with ^177^Lu-PSMA-617 and ^225^Ac-PSMA-617 but experienced progression or limited therapeutic response. Using radiation dose analysis software, SPECT imaging data were processed to compare absorbed doses in tumor lesions and dose-limiting organs between ^177^Lu-PSMA-617 and ^161^Tb-PSMA-617. The calculated therapeutic indices were 1.18 for the kidneys, 1.10 for the parotid glands, and 2.40 for tumor lesions. Compared with ^177^Lu-PSMA-617, ^161^Tb-PSMA-617 delivered higher absorbed doses to tumors with only marginal increases in organ doses, supporting ^161^Tb as a promising therapeutic radionuclide for PRLT and warranting larger prospective trials (47). A retrospective study conducted at the King Hussein Cancer Centre evaluated the safety and efficacy of ^161^Tb-PSMA-617 and ^177^Lu-PSMA-617 in heavily pre-treated patients with mCRPC who had undergone prior surgery, ADT, chemotherapy, and radiotherapy. Across 148 therapy cycles administered to 53 individuals (144 cycles of ^177^Lu-PSMA-617 and four cycles of ^161^Tb-PSMA-617), approximately half (n = 26) achieved a partial response, and one-quarter (n = 13) maintained a favorable biochemical response. Only 18 individuals (34%) experienced mild, self-limiting adverse effects (48).

Abdlkadir et al. (49) reported a case of dual-radionuclide therapy in a patient with mCRPC resistant to ^177^Lu-PSMA therapy. Combined administration of ^161^Tb-PSMA and ^177^Lu-PSMA resulted in marked reductions in serum PSA and tumor burden, suggesting a potential synergistic effect of this dual-isotope regimen. Chirindel et al. (50) evaluated the novel radiopharmaceutical ^161^Tb-SibuDAB in a patient with mCRPC and compared it with ^177^Lu-PSMA-I&T. The results demonstrated that ^161^Tb-SibuDAB delivered higher tumor doses and exhibited a longer effective half-life, supporting its value in targeting micrometastatic lesions. Importantly, no adverse reactions were reported.

Buteau et al. (51) assessed the safety and efficacy of ^161^Tb-PSMA-I&T in patients with mCRPC. No dose-limiting toxicities occurred, and only two participants (7%) experienced grade 3 treatment-related adverse events. There were no dose reductions, treatment delays, or treatment-related deaths. The PSA50 response rate was 70%, the PSA90 response rate was 40%, the median PSA progression-free survival was 9 months, and the median radiographic progression-free survival was 11 months. Organ dosimetry analyses showed that absorbed doses to normal tissues remained within accepted safety thresholds. Mean absorbed doses (Gy/GBq) were 0.15 for the parotid glands, 0.36 for the kidneys, 0.08 for the liver, and 0.06 for the spleen, confirming that radiation exposure to potential dose-limiting organs remained well within tolerable limits. Sezgin C et al. (38) reported the treatment of a 77-year-old patient with mCRPC who received two cycles of ^161^Tb-PSMA-617 treatment without adverse events; however, both PSA levels and imaging findings indicated disease progression. The authors also focused on temporal changes in activity levels and their implications for radiation safety. The mean external dose rate of ^161^Tb-PSMA-617 was 56 μSv/h at 6 h post-injection and decreased to 14 μSv/h at 24 h post-injection. These findings are valuable for establishing patient-isolation criteria and radiation-safety protocols. Kucuk et al. (52) enrolled seven patients with mCRPC who had progressed after at least 2 cycles of ^177^Lu-PSMA. Of the seven patients, four (57%) demonstrated objective imaging response and four (57%) showed at least a 50% decline in PSA levels. Treatment was well tolerated, with only mild adverse events (grades 1–2) and no toxicity greater than grade 3. Organ dosimetry confirmed favorable absorbed dose distributions. **Table 3** summarizes clinical studies of ^161^Tb-PRLT.

**Table 3:** Clinical studies of ^161^Tb-PRLT.

First author	Year	Country	Study type	Radiopharmaceutical	Patients	Administration protocol	Treatment response (based on PSA value)	Safety
Rosar et al. (45)	2023	Germany	Case report	^161^Tb-PSMA-617	One patient with ^177^Lu-PRLT failure	One cycle of ^161^Tb-PRLT (6.5 GBq)	PR (PSA decline by 53.4%, from 474 to 221 ng/mL)	No acute adverse effects or treatment-related adverse events
Al-Ibraheem et al. (46)	2023	Jordan	Case report	^161^Tb-PSMA-617	One patient	One cycle of ^161^Tb-PRLT (5.5 GBq)	Not provided	No acute or early adverse events
Schaefer-Schuler et al. (47)	2024	Germany	Retrospective study	^161^Tb-PSMA-617	Six patients with ^177^Lu-PRLT or ^225^Ac-PRLT failure	One cycle of ^161^Tb-PRLT per patient (6.4 ± 1.2 GBq; range, 5.1–8.7 GBq)	One patient (PR, PSA decline by 53.4%); three patients (SD, two patients PSA decline by 24.2% and 18.6%, one patient PSA increase by 18.0%); two patients (PD, PSA increase by 48.6% and 73.2%)	Manageable side effects (CTCAE, grade 1–3)
Al-Ibraheem et al. (48)	2024	Jordan	Retrospective study	^161^Tb-PSMA-617	Four patients with ^177^Lu-PRLT failure	One cycle of ^161^Tb-PRLT per patient (5.5 GBq)	One patient (PR, PSA decline by 69.8%); two patients (SD, PSA decline by 16.7% and 29.5%); one patient (PD, PSA increase by 71.3%)	Manageable side effects (CTCAE, grade 1–2)
Abdlkadir et al. (49)	2024	Jordan	Case report	^161^Tb-PSMA	One patient	One cycle of ^161^Tb-PRLT between two cycles of ^177^Lu-PRLT (5.5 GBq)	PR (PSA decline from 321 ng/mL to 27 ng/mL)	No side effects
Chirindel et al. (50)	2024	Switzerland	Case report	^161^Tb-SibuDAB	One patient	One cycle of ^161^Tb-PRLT (1 GBq)	No provided	No acute toxicity or remarkable adverse effects (CTCAE, grade 1–2)
Buteau et al. (51)	2025	Australia	Prospective study	^161^Tb-PSMA-I&T	Thirty patients	One to six cycles of ^161^Tb-PSMA-I&T per patient (three patients, 4.4 GBq; three patients, 5.5 GBq; 24 patients, 7.4 GBq)	Twenty one patients (PSA decline by more than 50%); Twelve patients (PSA decline by more than 90%); Five patients (PSA increase)	No radiation dose reductions, delays, or treatment-related deaths (CTCAE, grade 1–3)
Sezgin C et al. (38)	2025	Turkey	Case report	^161^Tb-PSMA-617	One patient	Two cycles of ^161^Tb-PSMA-617 (6.3 GBq, 7.6 GBq	PD (PSA increased from a of 4.3 ng/mL to 11.1 ng/mL)	No adverse events
Kucuk et al. (52)	2025	Turkey	Prospective study	^161^Tb-PSMA-I&T	Seven patients with ^177^Lu-PRLT failure	Two cycles of ^161^Tb-PSMA-I&T per patient (7.4 GBq)	Four patients (PSA decline by more than 50%); One patient (PSA decline by 2.7%); two patients (PSA increased)	Only mild adverse events (grades 1–2) and no toxicity greater than grade 3

PSA, prostate-specific antigen; PSMA, prostate-specific membrane antigen; PRLT, PSMA-targeted radioligand therapy; PR, partial remission; SD, stable disease; PD, progressive disease; CTCAE, Common Terminology Criteria for Adverse Events.

Several clinical trials are currently underway to evaluate the safety and efficacy of ^161^Tb-labelled PSMA ligands. The Australian VIOLET trial (NCT05521412) (53), a Phase I/II study investigating ^161^Tb-PSMA-I&T in patients with mCRPC, has released preliminary data (refer to the preceding section and **Table 3** for details). Additional studies on ^161^Tb-PSMA-617 with similar objectives are in progress in Germany (REALITY trial, NCT04833517). The Swiss PROGNOSTICS trial (NCT06343038) is a dose-escalation Phase Ia/b study evaluating ^161^Tb-SibuDAB for PRLT in patients with mCRPC. In China, an early-phase clinical trial (NCT06827080) assessing the safety, tolerability, and efficacy of ^161^Tb-NYM032 in patients with mCRPC is ongoing. **Table 4** provides an overview of the ongoing clinical trials investigating ^161^Tb-PRLT.

**Table 4:** Clinical trials of ^161^Tb-PRLT.

NCT identifier	Radiopharmaceutical	Start time	Country	Phase	Purpose
NCT04833517	^161^Tb-PSMA-617	2016-01	Germany	I	To assess outcome and toxicity in patients with mCRPC
NCT05521412	^161^Tb-PSMA-I&T	2022-09	Australia	I/II	To evaluate the efficacy and safety in patients with mCRPC
NCT06343038	^161^Tb-SibuDABs	2024-02	Switzerland	Ia/b	To evaluate the safety and efficacy in patients with mCRPC
NCT06827080	^161^Tb-NYM032	2025-02	China	I	To evaluate the safety, tolerability, and efficacy in patients with mCRPC

mCRPC, metastatic castration-resistant prostate cancer.

## 8 Discussion

Over the past decade, ^177^Lu-PRLT has achieved remarkable success in the treatment of mCRPC. In the VISION trial (20), ^177^Lu-PSMA-617 combined with standard care significantly improved median overall survival compared with standard care alone (15.3 months vs. 11.3 months), achieving a 38% reduction in mortality risk. The study also found a remarkable improvement in radiographic progression-free survival (8.7 months vs. 3.4 months), corresponding to a 60% reduction in the risk of radiographic progression or death. The U.S. Food and Drug Administration and the European Medicines Agency have approved ^177^Lu-PSMA-617 for the treatment of adults with PSMA-positive mCRPC following prior ADT and chemotherapy. ^177^Lu-PRLT demonstrates a favorable safety profile, prolongs survival, and improves quality of life in patients with mCRPC (54–56).

However, with successive treatment cycles, approximately 17%–30% of patients develop resistance to ^177^Lu-PRLT, resulting in disease progression (57). This resistance may be partly attributed to the physical characteristics of β^-^ particles, which exhibit medium-to-high energy and low LET. These particles predominantly induce single-strand DNA breaks and dispersed double-strand breaks, leading to relatively modest cytotoxicity. Furthermore, the penetration range of β^-^ particles occurs at the tissue scale, approximately 2 mm for ^177^Lu. Although this provides broad radiation coverage and is advantageous for treating macroscopic tumor volumes, the range exceeds the dimensions of microlesions. Consequently, a substantial portion of the emitted energy is deposited outside the target region, reducing the effectiveness of microlesion eradication. Furthermore, persistent microlesions may compromise long-term disease control (58).

^161^Tb has been recognized as a promising novel medical radioisotope with the potential to replace or complement ^177^Lu therapy. ^161^Tb exhibits physical characteristics similar to those of ^177^Lu, with both isotopes capable of emitting β^-^ particles. However, ^161^Tb provides an additional advantage through the emission of internal conversion electrons and Auger electrons, enabling the effective elimination of microlesions. These electrons have an ultra-short tissue range resulting in a high LET providing higher local dose densities. Both Monte Carlo simulations and preclinical studies have demonstrated that ^161^Tb-PSMA delivers a significantly higher radiation dose to tumor lesions and exhibits superior therapeutic efficacy compared to ^177^Lu-PSMA. Limited clinical studies have also confirmed the favorable therapeutic efficacy of ^161^Tb-PSMA. Meanwhile, the safety profile of ^161^Tb-PSMA is also encouraging. The primary adverse events are hematological toxicities, with some patients reporting dry mouth, fatigue, and nausea. Overall, ^161^Tb-PSMA is well-tolerated, with most adverse events being mild (grade 1-2). Grade 3 adverse events were uncommon, and no grade 4 adverse events were reported. However, clinical studies on ^161^Tb-PSMA remain limited, and real-world data are scarce. The currently published literature consists predominantly of case reports, with a lack of large-scale prospective studies. Large-scale, prospective cohort studies are essential to further evaluate critical clinical endpoints, such as safety, therapeutic efficacy, and treatment durability, and to establish robust, standardized criteria for assessing radiopharmaceutical treatment response.

Additionally, the successful translation of ^161^Tb from preclinical to clinical use largely depends on isotope availability. Like other reactor-produced radionuclides, ^161^Tb production faces key challenges, including restricted reactor capacity and logistical barriers to downstream application. As theranostic radionuclides continue to gain clinical prominence, the imbalance between production and global demand is expected to become increasingly evident. Furthermore, as an emerging isotope, ^161^Tb requires harmonized production protocols, secure supply chain management, and GMP-compliant quality control systems to ensure consistent radiochemical purity, safety, and efficacy. Fortunately, the close physicochemical similarity between ^161^Tb and ^177^Lu allows existing infrastructure, logistics, operational procedures, and radiation safety protocols used for ^177^Lu-labelled agents to be readily adapted for ^161^Tb-based compounds. This operational compatibility facilitates knowledge transfer from established ^177^Lu experience and supports the streamlined clinical adoption of ^161^Tb-labelled therapeutics.

## 9 Conclusion

This review provides a comprehensive synthesis of the mechanism of action, radiolabeling and quality control, dosimetry, preclinical evaluation, and clinical investigations of ^161^Tb-PSMA. Overall, ^161^Tb-labelled RLT—exemplified by ^161^Tb-PSMA—demonstrates considerable therapeutic promise. Continued research aimed at improving isotope accessibility, standardization, and clinical translation remains essential to fully realize its potential.
